# Overexpression of LncRNA MNX1-AS1/PPFIA4 Activates AKT/HIF-1*α* Signal Pathway to Promote Stemness of Colorectal Adenocarcinoma Cells

**DOI:** 10.1155/2022/8303409

**Published:** 2022-10-03

**Authors:** Qi Sun, Zhifu Gui, Zhenguo Zhao, Wenlong Xu, JunJia Zhu, Chundong Gao, Wei Zhao, Hao Hu

**Affiliations:** ^1^Department of General Surgery, The First Affiliated Hospital of Soochow University, Suzhou, Jiangsu Province 215006, China; ^2^Department of General Surgery, Jiangyin People's Hospital Affiliated to Nantong University, No. 163 Shoushan Rd, Jiangyin, Jiangsu Province 214400, China; ^3^Department of Clinical Biochemistry, School of Laboratory Medicine, Chengdu Medical College, No. 783, Xindu Rd., Chengdu, Sichuan 610500, China

## Abstract

**Purpose:**

The purpose of this study was to explore the role of the lncRNA MNX1-AS1 and its related downstream signaling pathways in colorectal adenocarcinoma (COAD).

**Methods:**

COAD tissues and cells were prepared and treated with sh-MNX1-AS1, pcDNA-MNX1-AS1, sh-PPFIA4, LY29004, and their controls. CCK8 and colony formation assays were undertaken for evaluating cell proliferation. Tumor cell migratory ability was detected by transwell assay. Apoptosis detection was processed by YO-PRO-1/PI Staining. The regulated relationship between lncRNA MNX1-AS1 and PPFIA4 was confirmed by RIP-ChIP assay. Q-PCR was applied to detect genes related to tumor cell stemness, proliferation, migration, and apoptosis in each group. Finally, a xenograft tumor model was constructed to verify the result *in vivo*.

**Results:**

COAD patients with high expression of the lncRNA MNX1-AS1 have poor prognosis. LncRNA MNX1-AS1 promotes the stemness of COAD cells. PPFIA4 mediates lncRNA MNX1-AS1 expression and affects COAD cell stemness. LncRNA MNX1-AS1 accelerates proliferation and migration, while it suppresses apoptosis. LncRNA MNX1-AS1/PPFIA4 accelerates tumor growth in COAD model. LncRNA MNX1-AS1/PPFIA4 activates the downstream AKT/HIF-1*α* signaling pathway to promote COAD development. LY29004 significantly inhibits the tumorigenic ability of lncRNA MNX1-AS1 and PPFIA4.

**Conclusion:**

LncRNA MNX1-AS1/PPFIA4 activates AKT/HIF-1*α* signal pathway to promote the stemness of COAD cells, which could be a new target for COAD treatment.

## 1. Introduction

Colorectal cancer (CRC) is a common malignant tumor with increasing morbidity and mortality [1]. According to the 2020 global cancer statistics, there are 555,000 new cases of CRC in China, ranking the third among malignant tumors [2]. The latest statistics from the National Cancer Center show that the number of new cases of CRC in China accounts for 9.9% of all new malignancies [3]. Colorectal adenocarcinoma (COAD) is the most common type of CRC [4]. The diagnosis of colorectal cancer involves imaging evaluation, pathological evaluation, and endoscopy, and the treatments are mainly surgery, chemotherapy, and radiotherapy [5]. Multidisciplinary comprehensive treatment model improves the level of colorectal cancer diagnosis and treatment.

With the continuous development of biotechnology, lncRNA has been identified to be a common molecular mechanism associated with tumors [6, 7]. Several lncRNAs with critical roles in tumor progression have been identified in colorectal cancer [8]. For instance, LncRNA NEAT1 increases its stability by activating the AKT signaling pathway, which in turn promotes colorectal cancer progression [9]. Furthermore, downregulation of LncRNA HOXD-AS1 promotes cell proliferation and metastasis by reducing the repressor marker (H3K27me3) of the HOXD3 promoter region in COAD cells [10]. Besides, lncRNA MEG3 increases the sensitivity of colorectal cancer cells to oxaliplatin by targeting miRNA-141 to upregulate PDCD4 expression [11]. MIR100HG has also been shown to be a potent EMT inducer in colorectal cancer, possibly contributing to cetuximab resistance and metastasis by activating the MIR100HG/hnRNPA2B1/TCF7L2 feedback loop [12]. Therefore, the potential of lncRNAs as novel biomarkers for the diagnosis and treatment of colorectal cancer cannot be ignored. Development of novel biomarkers not only improves treatment efficiency, but also provides more personalized treatment plans for COAD patients.

In recent years, lncRNA MNX1-AS1 was widely reported to be a cancer-promoting factor in various cancers, such as intrahepatic cholangiocarcinoma [13], ovarian cancer [14], and gastric cancer [15].

Cancer stem cells are the key to tumor self-maintenance, and its main sources are cancer progenitor cells [16, 17]. The genetic, epigenetic, and proliferation and division patterns of cancer stem cells regulate tumor proliferation and malignancy. Moreover, stem cells affect cancer metastasis by regulating EMT-related pathways [18]. Thereby, online databases, *in vitro* and *vivo* experiments were combined to research the role of lncRNA MNX1-AS1, its' downstream, and related pathways in COAD.

PPFIA4 was reported to be the target of lncRNA MNX1-AS1. A bioinformatic analysis was performed by Fu et al. [19] and confirmed that the mRNA level of PPFIA4 was higher in CRC tissue samples than that in normal colon tissue. In 2021, Huang et al. confirmed that PPFIA4 enhanced colorectal cancer glycolysis, which in turn promoted cell proliferation and migration [20]. In addition, MNX1-AS1/PPFIA4 activates the downstream AKT/HIF-1*α* pathway to promote COAD development in this study. Phosphorylation of AKT regulates cell growth, proliferation, and glucose metabolism [21]. Moreover, the target genes of HIF-1*α* have been referred to be involved in energy metabolism and angiogenesis of cells [22]. In this study, the role and mechanism of lncRNA MNX1-AS1 in the occurrence and development of COAD were investigated. The regulated relationship between lncRNA MNX1-AS1/PPFIA4 and AKT/HIF-1*α* pathway was explored. The results of this study may provide a theoretical basis for COAD treatment.

## 2. Materials and Methods

### 2.1. Database Analysis

The expression of MNX1-AS1 in COAD tissues was analyzed in 2 independent online TCGA databases, Gene Expression Profiling Interactive Analysis (GEPIA) [23] and The University of ALabama at Birmingham CANcer data analysis Portal (UALACN) [24]. The Starbase database [25] was applied to analyze the relationship between MNX1-AS1 expression and COAD prognosis.

### 2.2. Collection of Patient Tissues and Cell Treatment

Fifty patients with colorectal adenocarcinoma who underwent surgical resection in Department of General Surgery, Jiangyin People's Hospital Affiliated to Nantong University from April 2019 to May 2020 were collected. No radiotherapy, chemotherapy, or other tumor-specific treatments were received before surgery, and tumor tissues were collected from non-necrotic sites of the tumor. The study has been approved by the ethics committee of Jiangyin People's Hospital Affiliated to Nantong University (CD2019F13). The patients included 27 males and 23 females; the age ranged from 32 to 78 years old. All specimens were confirmed by postoperative pathological sections. Fresh surgical specimens of paracancerous and normal tissues were immediately cryopreserved. All patients were informed and gave their consents.

Various COAD cell lines (HT29, HCT116, T84, SW480, LOVO, and SW620) and HCM460 cell line were purchased from ATCC, subcultured in RPMI1640 medium containing 10% FBS at 37 °C and 5% CO_2_-saturated humidity. Cells in logarithmic growth phase were taken for experiments. Suspension sphere culture was used to screen stem cells in HT29 and HCT116 cells. Briefly, tumor cells were incubated in a serum-free medium supplemented with growth factors, and stem cells therein proliferated to form dense spheres and maintain an undifferentiated state.

HT29, HCT116, HT29 CSC, and HCT116 CSC cells were made into cell suspension and divided into shRNA-NC group and sh-MNX1-AS1 group. Referring to the instructions of the kit, Lipofectamine® 2000 was used to transfect sh-MNX1-AS1 and its control into cells, and the cells were cultured for 48 h. Cells were collected for RT-PCR detection of MNX1-AS1 expression levels in cells of each group. Sphere formation was observed and photographed by inverted microscope. Similarly, Bio-MNX1-ASA, pcDNA-MNX1-AS1, sh-PPFIA4, and their controls were also transfected based on the above steps. For pathway exploring, PI3K inhibitor LY29004 (LY, 20 mM) was used to treat cells in different groups. The same number of transfected tumor stem cells in each group were taken, seeded in 6-well plates, and treated with LY29004 for 48 h.

### 2.3. Q-PCR Assay

Total RNA from tissues and cells was isolated by TRIzol reagent. The total RNA was reversely transcribed according to the instructions of the cDNA reverse transcription kit, and the cDNA was used as the template. Amplification was performed according to the instructions of the Fast Start Universal SYBR Green Mastermix kit. The reaction was carried out for 40 cycles, and the conditions were predenaturation at 95 °C for 5 s, denaturation at 95 °C for 5 s, and annealing at 60 °C for 30 s. The relative expression of MNX1-AS1 was calculated by 2^-*ΔΔ*^Ct method with GAPDH as internal reference. Besides, stemness markers (OCT4, CD33, CD133, and SOX2), proliferation-related genes (cyclin A1 and B1), migration-related genes (E-cadherin and N-cadherin) and apoptosis-related genes (Bax and Bcl-2) were also detected by Q-PCR. Primers were designed as follows: MNX1-AS1, (F) 5′-CCCGCATTTTCAGATTCAC-3′, (R) 5′-GCTCTCAGCCTCGCCATA-3′; GAPDH, (F) 5′-GTCAACGGATTTGGTCTGTATT-3′, (R) 5′ -AGTCTTCTGGGTGGGCAGTGAT-3′, OCT4, (F) 5′-CCCCAATGCCGTGAAGTT-3′, (R) 5′-GAAAGGTGTCCCTGTAGCG-3′; CD33, (F) 5′-ATCCGCCTAAACTCACATGG-3′, (R) 5′-AGGAATGAACGTCAGGATGG-3′; CD133, (F) 5′-TTCTGCCTGTGTAACTTTGCA-3′, (R) 5′-TTGTTGTGCAACGTCTTTGCA-3′; SOX2, (F) 5′-CGAGATAAACATGGCAATCAAAAT-3′, (R) 5′-AATTGAGAAGCCTCTCCTT-3′; cyclin A1, (F) 5′-GCTTTCCCGCAATCATGTACCC-3 ′, (R) 5 ′-CTCAAATGCCATCCCCTCTCT-3′; cyclin B1, (F) 5′-GCCAATAAGGAGGGAGCA-3′, (R) 5′-AGCGGGGAGAAGCAGAAC-3′; E-cadherin, (F) 5′-TTACTGCCCCCAGAGGATGA-3′, (R) 5′-TGCAACGTCGTTACGAGTCA-3′; N-cadherin, (F) 5′-GACAATGCCCTCAAGTGTT-3′, (R) 5′-CCATTAAGCCGAGTGATGGT-3′; Bax, (F)5′-GTGAATGGAGCCACTGCGCA-3′, (R) 5′-CCCCATCCCGGAAGAGTTCA-3′; Bcl-2, (F) 5′-GGTGCCACCTGTGGTCCACCTG-3′, (R) 5′-GTTCACTGTTGGCCCCAGATAGG-3′.

### 2.4. CCK8 Assay

A total of 100 ml of cell suspension with 2 × 10^3^ cells per well were added in a 96-well plate. Three replicate wells were set in each group, and the culture plates were pre-incubated in an incubator for 24 h at 37 °C and 5% CO_2_. 10 ml of cells in the transfection group and control group were added to the culture plate. After the culture plate was incubated in the incubator, 10 mL of CCK-8 solution was added to each well, and the culture plate was further incubated in the incubator for 2 h. The cells were taken for CCK-8 assay at 12 h, 24 h, and 48 h after transfection, and their absorbance was measured at a wavelength of 450 nm using an enzyme-linked immunosorbent assay.

### 2.5. Colony Formation

After transfection and treatment, cells were seeded in 6-well plates at a density of 200 cells per well, and three duplicate wells were set up. At the same time, 2 mL of 1640 medium containing 10% FBS was added to each well and cultured in an incubator at 37 °C, 5% CO_2_, and 95% saturated humidity for 2 to 3 weeks. When macroscopic clones appeared in the Petri dish, the culture was terminated, the supernatant was discarded, and the cells were carefully washed twice with PBS. Cells were fixed in 5 ml of 4% paraformaldehyde for 15 min. Then, the fixative solution was removed, and an appropriate amount of GIMSA staining solution was added to the sample for staining for 20 min. Then, the staining solution was slowly washed away with running water and dried in air. The number of clones greater than 50 cells was counted under the microscope.

### 2.6. Transwell Assay

The cells were digested with trypsin, washed twice with sterile phosphate buffer solution, and resuspended in 10 g/L FBS free medium to adjust the cell density to 1 × 10^5^ cells/ml. A total of 150 *μ*l of cell suspension was added to the upper chamber of the chamber precovered with Matrigel, and 500 *μ*L of medium containing 200 ml/L serum was added to the lower chamber. After 48 h of incubation, the chamber was removed and rinsed with sterile PBS, and the cells in the inner layer of the microporous membrane were carefully wiped off with a cotton swab. Cells were fixed with 95% ethanol for 6 min, stained with 4 g/L crystal violet solution, counted, and photographed under an inverted microscope. The average value of 5 visual fields was randomly selected to analyze the differences between groups.

### 2.7. Apoptosis Detection by YO-PRO-1/PI Staining

Cells were trypsinized and washed twice with precooled PBS. A mixed solution of 0.5 ml YP/PI was added, the final concentration of YP was 1 *μ*mol/L, and the final concentration of PI was 2 *μ*g/ml. The percentage of apoptotic cells in about 200 cells was counted under a fluorescence microscope after 15 min of reaction in the dark at room temperature. Each treatment was repeated 3 times.

### 2.8. RIP-ChIP Assay

The experiment was performed according to the instructions of the RIP test kit. Cells were grown to 90% confluence. The cell suspension was prepared, and RIP buffer was added. Anti-Bio or anti-PPFIA4 was incubated with cell suspension overnight at 4 °C. Proteinase K digestion was added. The precipitated PPFIA4 was analyzed by Western blot, using isotype IgG as a negative control and input as a total protein control.

### 2.9. Western Blot Assay

The total protein in the samples was extracted separately. Protein samples were prepared and run on SDS-PAGE gels and then transferred to PVDF membranes. The blocking solution containing 5% BSA was added and blocked for 2 h at room temperature. An appropriate concentration of primary antibody was added and blocked overnight at 4 °C. The next day, the PVDF membrane was washed three times with buffer, and the secondary antibody was added. After incubating for 1 h at room temperature, the chromogenic solution was added for exposure and development.

### 2.10. Construction of Xenograft Mode

Total 12 BALB/C nude mice were reared adaptively for 1 week and randomly divided into 4 groups (control, MNX1-AS1, MNX1-AS1 + sh-PPFIA4, and sh-PPFIA4 groups). Cells in each group were collected and adjusted to a cell concentration of 5 × 104/ml. 0.2 ml of cells were inoculated into the right armpit of BALB/C nude mice with a microsyringe and observed every 2 days to record the tumor formation in nude mice. Indications such as tumor nodules and hard texture at the site to be inoculated were identified as tumor formation. Vernier calipers were used to measure the largest diameter (length) and the two smallest diameters (width) of the transplanted tumor. The formula for calculating tumor volume is as follows: tumor volume (mm^3^) = length × width × width × 0.52. This experiment was approved by the Animal Ethics Committee of Chengdu Medical College.

### 2.11. Statistical Analysis

SPSS 20.0 software was used for statistical analysis. If the measurement data were normally distributed, they were described as the mean ± standard deviation. For measurement data with normal distribution and homogeneous variance, *t*-test or one-way comparison analysis was used. *P* < 0.05 was regarded as statistically significant.

## 3. Results

### 3.1. LncRNA MNX1-AS1 Expressed at High Level in COAD Tissues

**Figure 1:**
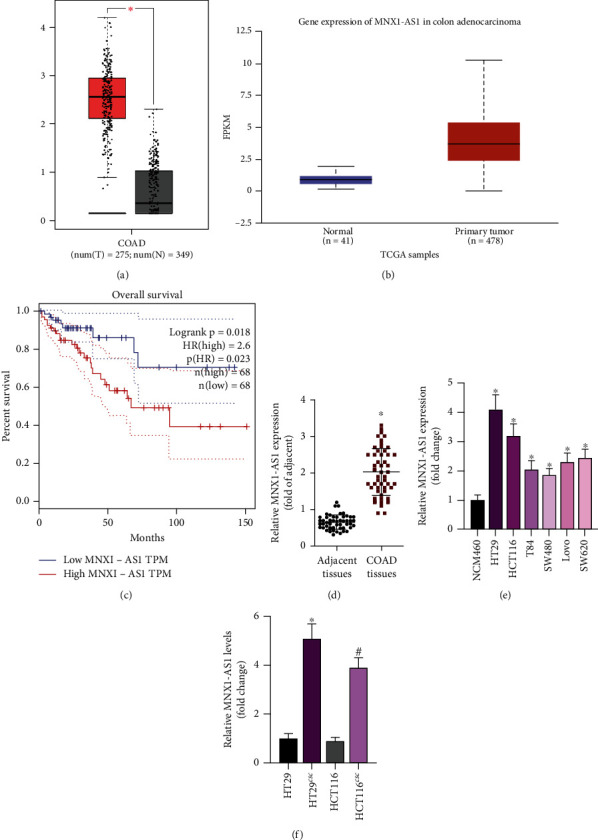
MNX1-AS1 is highly expressed in colorectal cancer tissues. Base on GEPIA (a) and UALACN (b) databases, analysis of MNX1-AS1 expression in COAD tissues. (c) Starbase database analysis of the relationship between MNX1-AS1 expression and COAD prognosis. (d) qPCR detection for MNX1-AS1 expression in COAD tissues, ^∗^*P* < 0.05 compared with Adjacent tissues. (e) qPCR detection for MNX1-AS1 expression in colorectal cancer tissues, ^∗^*P* < 0.05 compared with NCM460. (f) qPCR detection for MNX1-AS1 expression in cancer stem cells, ^∗^*P* < 0.05 compared with H29, ^#^*P* < 0.05 compared with HCT116.

The expression of lncRNA MNX1-AS1 was analyzed by 2 online databases and detected in cells and patients' tissues. Based on TCGA databases, lncRNA MNX1-AS1 expressed higher in COAD tissue compared to normal tissues (Figures 1(a) and 1(b)). Moreover, Starbase database was used to detect the relationship betweenMNX1-AS1 expression and COAD prognosis. The patients were divided into 2 groups based on the median value of lncRNA MNX1-AS1. As is shown in Figure 1(c), patients with high ncRNA MNX1-AS1 expression were associated with poor overall survival. Then, the level of lncRNA MNX1-AS1 was detected in COAD tissue, COAD cells, and COAD CSC cells. The results were similar in the data of TCGA and Starbase databases. LncRNA MNX1-AS1 is expressed at a high level in COAD tissues and cells (Figures 1(d) and 1(e)). Among these COAD cell lines, HTC29 and HCT116 were with the highest lncRNA MNX1-AS1 level, which was used for further experiments. Interestingly, lncRNA MNX1-AS1 level in SCS group was higher than that in the corresponding COAD cell lines (Figure 1(f)). According to these results, lncRNA MNX1-AS1 was critical in COAD development, which also affects the stemness of COAD.

### 3.2. Sh-MNX1-AS1 Inhibited Stemness of COAD Stem Cells

**Figure 2:**
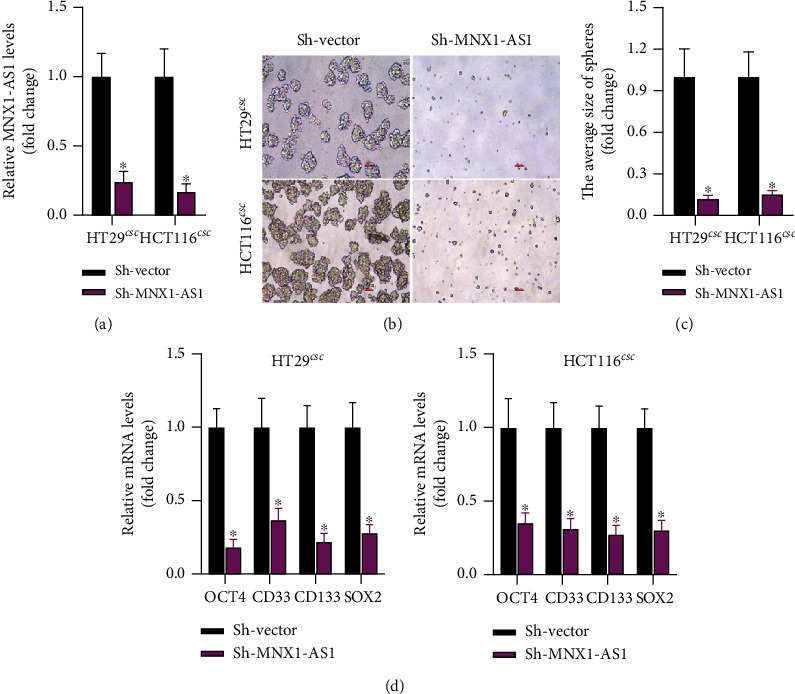
Knockdown of MNX1-AS1 inhibits the stemness of COAD cells. (a) MNX1-AS1 expression in COAD stem cells. (b, c) Microscopic observation of spheroid formation. (d) QPCR detection of tumor stemness markers, ^∗^*P* < 0.05 compared with sh-Vector.

Sh-MNX1-AS1 was transfected into COAD SCS to explore its' function. After sh-MNX1-AS1 transfection in HT29 CSC and HCT116 CSC cells, the level of MNX1-AS1 was clearly lower than that in sh-Vector group, indicating that the transfection was successful (Figure 2(a)). Moreover, the sizes of the spheres were photographed and calculated. As is shown in Figures 2(b) and 2(c), sphere formation was significantly inhibited by sh-MNX1-AS1. Then, the stemness of COAD CSC related genes was detected by q-PCR. As we expected, sh-MNX1-AS1 decreased the levels of OCT4, CD33, CD133, and SOX2 (Figure 2(d)). These results confirmed that sh-MNX1-AS1 suppressed the stemness of COAD stem cells.

### 3.3. Sh-MNX1-AS1 Suppressed Proliferation and Migration, While Accelerated Apoptosis of COAD Cells

**Figure 3:**
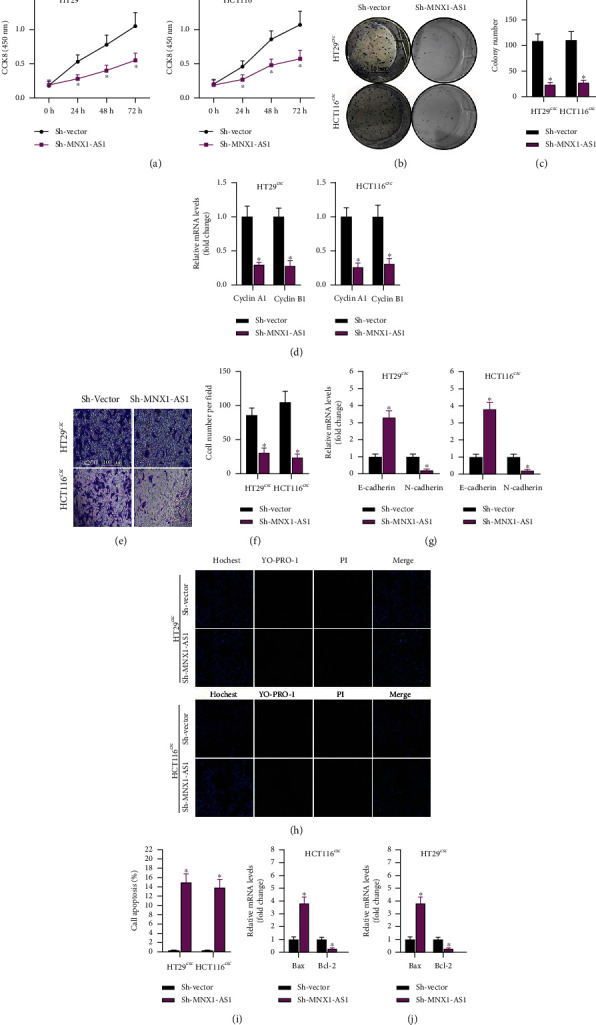
Effects of sh-MNX1-AS1 on COAD cell behavior. (a) The effect of sh-MNX1-AS1 on the proliferation of COAD stem cells detected by CCK8 assay. (b, c) Colony formation assay for detecting the proliferation of COAD cells. (d) RT-qPCR detection for expression of proliferation related genes. (e, f) Transwell assay for stem cell migration. (g) RT-qPCR detection of migration related gene expression. (h, i) YO-PRO-1/PI staining for detection of apoptosis. (j) RT-qPCR detected apoptotic gene expression. ^∗^*P* < 0.05 compared to sh-Vector.

The proliferation and apoptosis ability affected by sh-MNX1-AS1 transfection were explored. After 24 h, the proliferation was inhibited significantly. The difference between the two groups gradually increased over time (Figure 3(a)). Similarly, the colony number in sh-MNX1-AS1 was clearly lower than that in sh-Vector group (Figures 3(b) and 3(c)). Proliferation-related genes (cyclin A1 and B1) were also detected by Q-PCR. The results were consistent with CCK8 and colony formation. Both cyclin A1 and B1 expressed lower in HT29 SCS and HCT116 SCS (Figure 3(d)). Migration and its' related genes (E-cadherin and N-cadherin) were monitored. Migration was inhibited by sh-MNX1-AS1 (Figures 3(e) and 3(f)). Furthermore, E-cadherin was upregulated, while N-cadherin was increased (Figure 3(g)). Then, apoptosis and its' related genes (Bax and Bcl-2) were detected. As shown in Figures 3(h)–3(j), sh-MNX1-AS1 promoted apoptosis, and the tendency of Bax and Bcl-2 levels was consistent with the result of apoptosis. Therefore, sh-MNX1-AS1 inhibited the development of COAD SCS.

### 3.4. LncRNA MNX1-AS1 Upregulated PPFIA4 Expression

**Figure 4:**
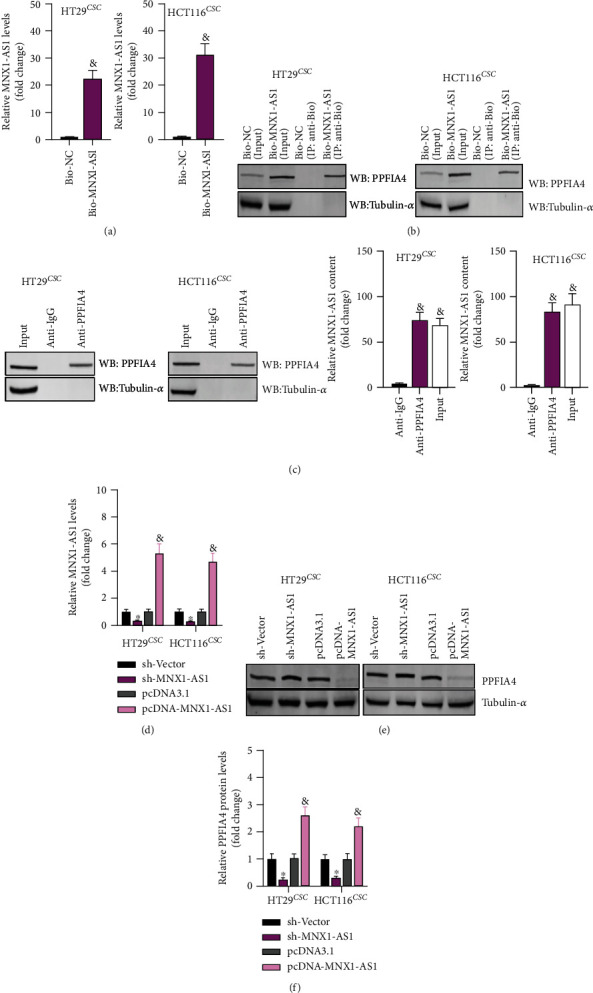
MNX1-AS1 targets and positively regulates PPFIA4. (a) The expression of biotin-labeled MNX1-AS1 in COAD stem cells. ^∗^*P* < 0.05 compared to Bio-NC. (b, c) Interaction between Bio-MNX1-AS1 and endogenous PPFIA4. ^&^*P* < 0.05 compared with anti-IgG. (d) Effect of knockdown or overexpression of MNX1-AS1 in COAD stem cells, ^∗^*P* < 0.05 compared with sh-Vector, and ^&^*P* < 0.05 compared with pcDNA3.1. (e, f) PPFIA4 protein expression, ^∗^*P* < 0.05 compared with sh-Vector, and ^&^*P* < 0.05 compared with pcDNA3.1.

Furthermore, the regulation relationship between lncRNA MNX1 and PPFIA4 was verified. RT-qPCR assay detected the expression of biotin-labeled (bio)-MNX1-AS1 in cancer stem cells. Relative lncRNA MNX1-AS1 level in bio-MNX1-AS1 was higher than that in control (Figure 4(a)). The interaction of Bio-MNX1-AS1 with endogenous PPFIA4 was detected by RIP-ChIP assay with anti-Bio or anti-PPFIA4. As shown in Figures 4(b) and 4(c), MNX1-AS1 interacted with PPFIA4 protein. After sh-MNX1-AS1 and pcDNA-MNX1-AS1 transfection, lncRNA MNX1-AS1 was regulated (Figure 4(d)), indicating that the transfection were successful. RT-qPCR and Western blot were used to examine the effect of knockdown or overexpression of lncRNA MNX1-AS1 in cancer stem cells. LncRNA MNX1-AS1 negatively regulated PPFIA4 expression (Figures 4(e) and 4(f)).

### 3.5. Knockdown of PPFIA4 Significantly Inhibits the Effect of lncRNA MNX1-AS1 on Stemness

**Figure 5:**
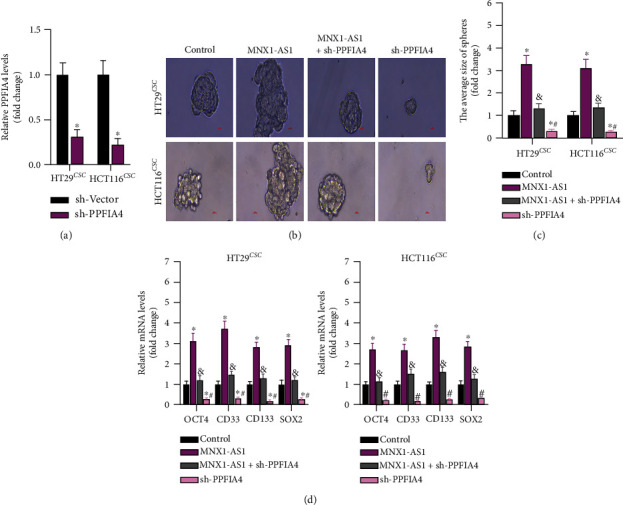
Knockdown of PPFIA4 significantly inhibits the effect of MNX1-AS1 on stemness of COAD cells. (a) Expression of PPFIA4, ^∗^*P* < 0.05 compared to sh-Vector. (b, c) Spheroid formation of COAD stem cells, ^&^*P* < 0.05 compared with MNX1-AS1 group, and ^#^*P* < 0.05 compared with MNX1-AS1 + sh-PPFIA4 group. (d) Expression of tumor stemness markers, ^∗^*P* < 0.05 compared with control group, ^&^*P* < 0.05 compared with MNX1-AS1 group, and ^#^*P* < 0.05 compared with MNX1-AS1 + sh-PPFIA4 group.

Then, the molecular mechanism of PPFIA4 in stemness was also researched. PPFIA4 significant inhibited PPFIA4 expression in HT29 and HCT116 CSCs, indicating that the transfection of sh-PPFIA4 was successful (Figure 5(a)). The spheroid formation of tumor stem cells was observed under the microscope. Interestingly, lncRNA MNX1-AS1 improved the spheroid forming ability, while sh-PPFIA4 transfection had negative effects (Figures 4(b) and 4(c)). RT-qPCR was used to detect the effect of lncRNA MNX1-AS1 and sh-PPFIA4 on the expression of tumor stemness markers. As shown in Figure 4(d), all stemness markers including OCT4, CD33, CD133, and SOX2 were upregulated by lncRNA MNX1-AS1, while they were decreased by sh-PPFIA4. These results showed that the stemness of COAD cells was clearly affected by lncRNA MNX1-AS1 and sh-PPFIA4.

### 3.6. PPFIA4 Suppressed the Effect of lncRNA MNX1-AS1 on the Behavior of COAD Stem Cells

**Figure 6:**
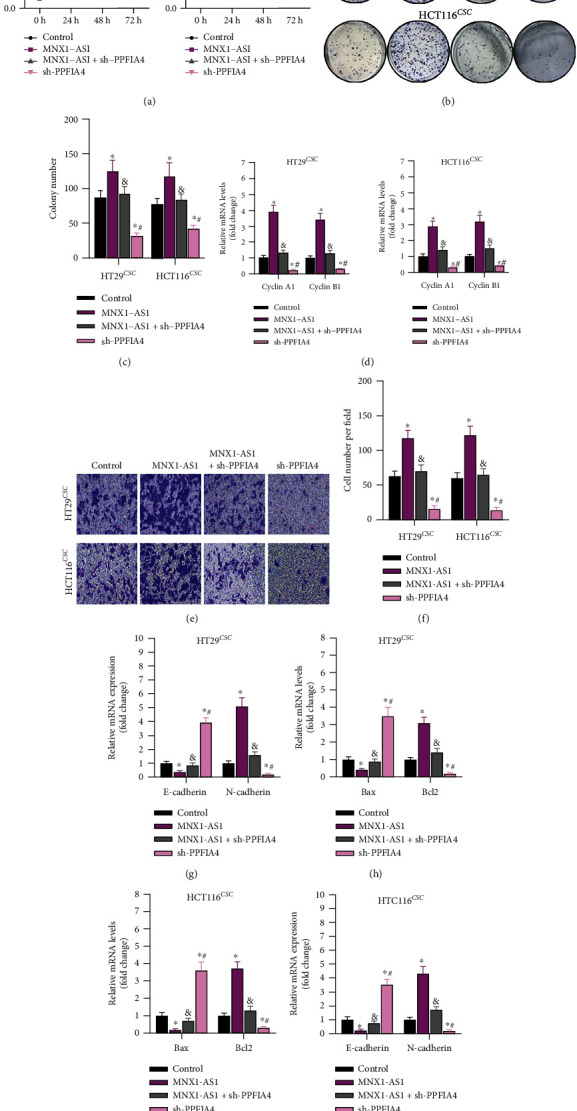
Knockdown of PPFIA4 rescues the effect of MNX1-AS1 on the behavior of COAD stem cells. (a) The effect of overexpression of MNX1-AS1 or knockdown of PPFIA4 on the proliferation of cancer stem cells. (b, c) Colony formation assay for detecting the proliferation. (d) Effects of overexpression of MNX1-AS1 or knockdown of PPFIA4 on expression of proliferation gene. (e, f) Cell migration. (g) Expression of cancer stem cell migration-related genes. (h, i) Effects of overexpression of MNX1-AS1 or knockdown of PPFIA4 on apoptosis of COAD stem cells. (j) Expression of apoptosis-related genes, ^∗^*P* < 0.05 compared with the control group, ^&^*P* < 0.05 compared with the MNX1-AS1 group, and ^#^*P* < 0.05 compared with the MNX1-AS1 + sh-PPFIA4 group.

The roles of LncRNA MNX1-AS1 and sh-PPFIA4 were further researched in COAD stem cells. Based on the transfection, the cells were divided into 4 groups, including control, MNX1-AS1, MNX1-AS1 + sh-PPFIA4, and sh-PPFIA4 groups. After 48 h, the cell proliferation was inhibited significantly in 3 groups (Figure 6(a)). Similarly, the colony number in MNX1-AS1 group was higher than controls, while sh-PPFIA4 inhibited the proliferation behavior (Figures 6(b) and 6(c)). Proliferation-related genes (cyclin A1 and B1) were also detected by Q-PCR. The results were consistent with CCK8 and colony formation. Both cyclin A1 and B1 were at a higher level in MNX1-AS1 group, and at the lowest level in sh-PPFIA4 group (Figure 6(d)). Migration was accelerated by MNX1-AS1, which was inhibited by sh-PPFIA4 (Figures 6(e) and 6(f)). Furthermore, E-cadherin was upregulated, while N-cadherin was increased after lncRNA MNX1-AS1 was upregulated (Figure 6(g)). Then, apoptosis and its' related genes (Bax and Bcl-2) were detected. As shown in Figures 6(h)–6(j), MNX1-AS1 inhibited apoptosis, and the tendency of Bax and Bcl-2 levels was consistent with the result of apoptosis. Sh-PPFIA4 rescued these effects. Therefore, sh-PPFIA4 transfection rescued the effect of upregulated lncRNA MNX1-AS1 on the behavior of COAD stem cells.

### 3.7. Sh-PPFIA4 Significantly Inhibited the Tumorigenic Effect of lncRNA MNX1-AS1

**Figure 7:**
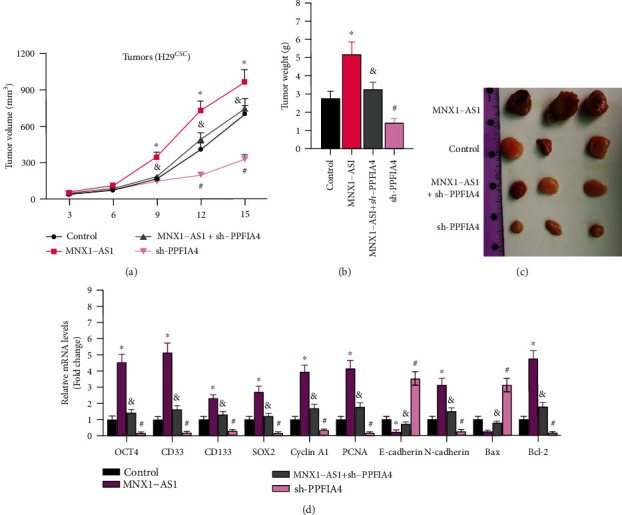
Knockdown of PPFIA4 inhibits the tumorigenic effect of MNX1-AS1. (a) The changes of tumor volume of each stably transfected cell line. (b) Changes in tumor weight. (c) Tumor photograph. (d) Expression of COAD cell stemness, proliferation, invasion and metastasis, and apoptosis-related genes in xenograft tumor tissues, ^∗^*P* < 0.05 compared with control group, ^&^*P* < 0.05 compared with MNX1-AS1 group, and ^#^*P* < 0.05 compared with MNX1-AS1 + sh-PPFIA4 group.

The roles of LncRNA MNX1-AS1 and sh-PPFIA4 in vivo were also detected. The changes of volume and weight of tumors in 4 groups were evaluated. As shown in Figures 7(a)–7(c), lncRNA MNX1-AS1 improved tumor growth, while sh-PPFIA4 decreased the tumorigenic ability. Q-PCR assay was used to detect the gene expression related to cancer cell stemness, proliferation, invasion and metastasis, and apoptosis in xenograft tissue. The results and tendency were consistent with the experiments in vitro (Figure 7(d)). These results suggested that sh-PPFIA4 significantly inhibited the tumorigenic effect of lncRNA MNX1-AS1 in vivo.

### 3.8. LncRNA MNX1-AS1/PPFIA4 Regulates the AKT/HIF-1*α* Signaling Pathway

**Figure 8:**
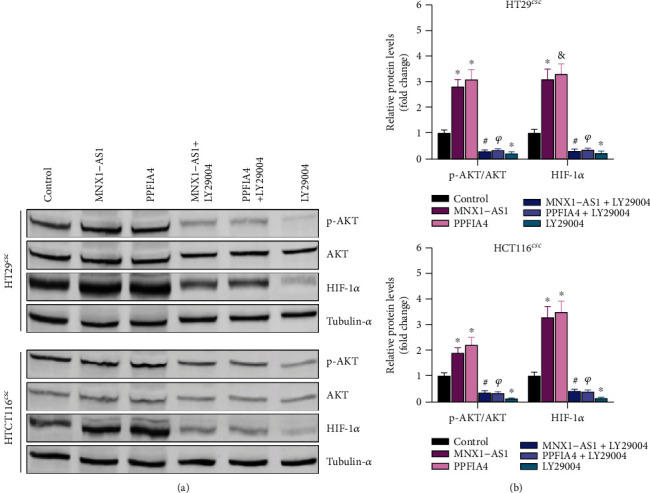
MNX1-AS1/PPFIA4 functions in COAD through the AKT/HIF-1*α* signaling pathway. (a, b) Expression of p-AKT, AKT, and HIF-1*α* proteins in the AKT/HIF-1*α* signaling pathway, ^∗^*P* < 0.05 compared with the control group, ^#^*P* < 0.05 compared with the MNX1-AS1 group, ^*ψ*^*P* < 0.05 compared with PPFIA4 group.

To detect the effect of lncRNA MNX1-AS1/PPFIA4 on AKT/HIF-1*α* signaling pathway, Western blot experiment was performed to detect the expressions of p-AKT, AKT, and HIF-1*α* proteins in the AKT/HIF-1*α* signaling pathway. The relative expressions of p-AKT/AKT and HIF1*α* were higher in MNX1-AS1 and PPFI4A groups, while they were lowest in LY20994 group (Figures 8(a) and 8(b)). These results indicated that lncRNA MNX1-AS1/PPFIA4 was involved in AKT/HIF-1*α* signaling pathway.

### 3.9. LY29004 Significantly Inhibited the Tumorigenic Ability of lncRNA MNX1-AS1 and PPFIA4

Finally, AKT-specific inhibitor (LY294002) was used to verify the effect of lncRNA MNX1-AS1/PPFIA4 on the proliferation and apoptosis of colorectal cancer *in vivo.* LY29004 significantly inhibited the tumorigenic ability of MNX1-AS1 and PPFIA4 (Supplementary Figures 1(a), 1(b), 1(d), and 1(e)). Q-PCR was used to detect the gene expression related to stemness, proliferation, migration, and apoptosis in each group in vivo. The results were consistent with the experiment in cells (Supplementary Figures 1(c) and 1(f)).

## 4. Discussion

The progression of colorectal cancer involves multiple genetic mutations. The most widely studied in recent years is the role of long noncoding RNAs in the growth and development of colorectal cancer [26, 27]. Some studies have demonstrated that lncRNA plays an important role in the stemness and growth of colorectal cancer cells [28]. In this study, MNX1-AS1 and its related downstream signaling pathways were researched in colorectal adenocarcinoma (COAD). Interestingly, COAD patients with high expression of MNX1-AS1 have poor prognosis. Moreover, MNX1-AS1 promotes stemness and proliferation of COAD cells.

The lncRNA MNX1-AS1 is located on chromosome 7, which can be transcribed to produce a product with 992 bps. MNX1-AS1 has been reported to promote cell proliferation and invasion in many malignancies [21, 29–31]. The expression of MNX1-AS1 in osteosarcoma tissues was higher than that in adjacent tissues [32]. The expression of MNX1-AS1 in osteosarcoma tissues was higher than that in adjacent tissues. Liu et al. reported that the high expression of MNX1-AS1 in tumor tissue in non-small cell lung cancer is closely related to tumor TNM stage and lymph node metastasis [22]. In this study, sh-MNX1-AS1 suppressed cell proliferation and migration, while it accelerated apoptosis. Importantly, MNX1-AS1 accelerated tumorigenic ability in *vivo*. Thereby, the role of MNX1-AS1 in COAD could not be ignored. Consistent with previous report [31], MNX1-AS1 is upregulated in COAD. Moreover, they also found that MYC, a high-frequent amplified oncogene, binds to the promoter of MNX1-AS1 and activated its transcription [31].

Furthermore, sh-MNX1-AS1 decreased the levels of OCT4, CD33, CD133, and SOX2 in this study, indicating that sh-MNX1-AS1 suppressed the stemness of COAD stem cells. In recent years, somatic cell reprogramming, dedifferentiation, and pluripotent stem cell origin have become the focus of stem cell research [33, 34]. Oct4 and Sox2 are the key core regulators for maintaining pluripotency and self-renewal of stem cells [35]. They can not only regulate the expression of multiple genes related to maintaining pluripotency, self-renewal, and multidirectional differentiation, but also participate in signaling transduction and epigenetics [36]. In addition, CD133 (Prominin-l) is one of the typical TSC surface markers, which is expressed in breast cancer, kidney cancer, colorectal cancer, and other malignant tumors [37]. It is currently considered a recognized TSC cell marker. Besides, CD33 induces rapid phosphorylation of tyrosine motifs [38]. When it is stimulated by an exogenous protein kinase, the cytoplasmic tail will undergo tyrosine phosphorylation, and then the immunoreceptor tyrosine-related inhibitory motif (ITIM) acts as a cell membrane molecule to transmit inhibitory signal into the cell. In this study, the expression of OCT4, CD33, CD133, and SOX2 were all inhibited by sh-MNX1-AS1 transfection, indicating that sh-MNX1-AS1 inhibited the stemness of COAD cells.

Moreover, the results in this study also confirmed that MNX1-AS1 targets and positively regulates PPFIA4. In previous studies, PPFIA4 was confirmed to enhance colorectal cancer glycolysis, which in turn promotes cell proliferation and migration [17] Li et al. [39] performed a comprehensive integration of metastatic colorectal cancer (mCRC) genomics, proteomics, and phosphoproteomics and referred that the PPFIA4 gene has a higher mutation frequency in CCRC. It was worth noting that PPFIA4 mediates MNX1-AS1 and affects COAD cell stemness in this study. Sh-PPFIA4 suppressed proliferation and migration, while it accelerated apoptosis of tumor cells. MNX1-AS1/PPFIA4 accelerated tumor growth in COAD model. LncRNA MNX1-AS1/PPFIA4 acted as a promoting factor of COAD through the AKT/HIF-1*α* pathway. The active region of Akt protein induces conformational changes by regulating the binding of Akt to PI-3, 4, 5-P3 [40]. Apoptosis of tumor cells includes two pathways, p53-dependent and p53-independent [41]. Some studies suggested that hypoxia-induced apoptosis worked through a HIF-1*α*-mediated 53-dependent pathway [42, 43]. HIF-1*α* provides energy to tumor cells, thereby enhancing tumor cell viability [44]. In pancreatic, colon, and breast cancer, HIF-1*α* was positively correlated with proliferating cell nuclear antigen [45–47]. Similar results were also obtained in this study. P-AKT/AKT and HIF1*α* expressed at a high level in MNX1-AS1 and PPFI4A groups, while they were lowest in LY20994 group. The result indicated that MNX1-AS1/PPFIA4 was involved in AKT/HIF-1*α* signaling pathway. Importantly, LY29004 significantly inhibited the tumorigenic ability of lncRNA MNX1-AS1 and PPFIA4 in *vivo*.

In conclusion, lncRNA MNX1-AS1/PPFIA4 activated AKT/HIF-1*α* signaling pathway to promote stemness of COAD cells, which could be a new target for COAD treatment.

## Data Availability

The original data presented in this study are included in the article/Supplementary Material, and further inquiries can be directed to the corresponding authors.

## Supplementary Materials

Supplementary Materials Suppl. Figure 1. LY29004 rescues MNX1-AS1 and PPFIA4 *in vivo*. (a, d) Nude mouse xenograft tumors. (b, e) After 15 days, the tumors were weighed. (c, f) Expression of tumor cell stemness, proliferation, migration, and apoptosis-related genes in tumor tissues of each group, ^∗^*P* < 0.05 compared with control group, and ^#^*P* < 0.05 compared with MNX1-AS1 group or PPFIA4 group.
