# Supplementary figures and images for: Overexpression of LncRNA MNX1-AS1/PPFIA4 Activates AKT/HIF-1α Signal Pathway to Promote Stemness of Colorectal Adenocarcinoma Cells
Source: J Oncol. 2022 Oct 3;2022:8303409. doi: 10.1155/2022/8303409 (PMC9550508; doi:10.1155/2022/8303409)

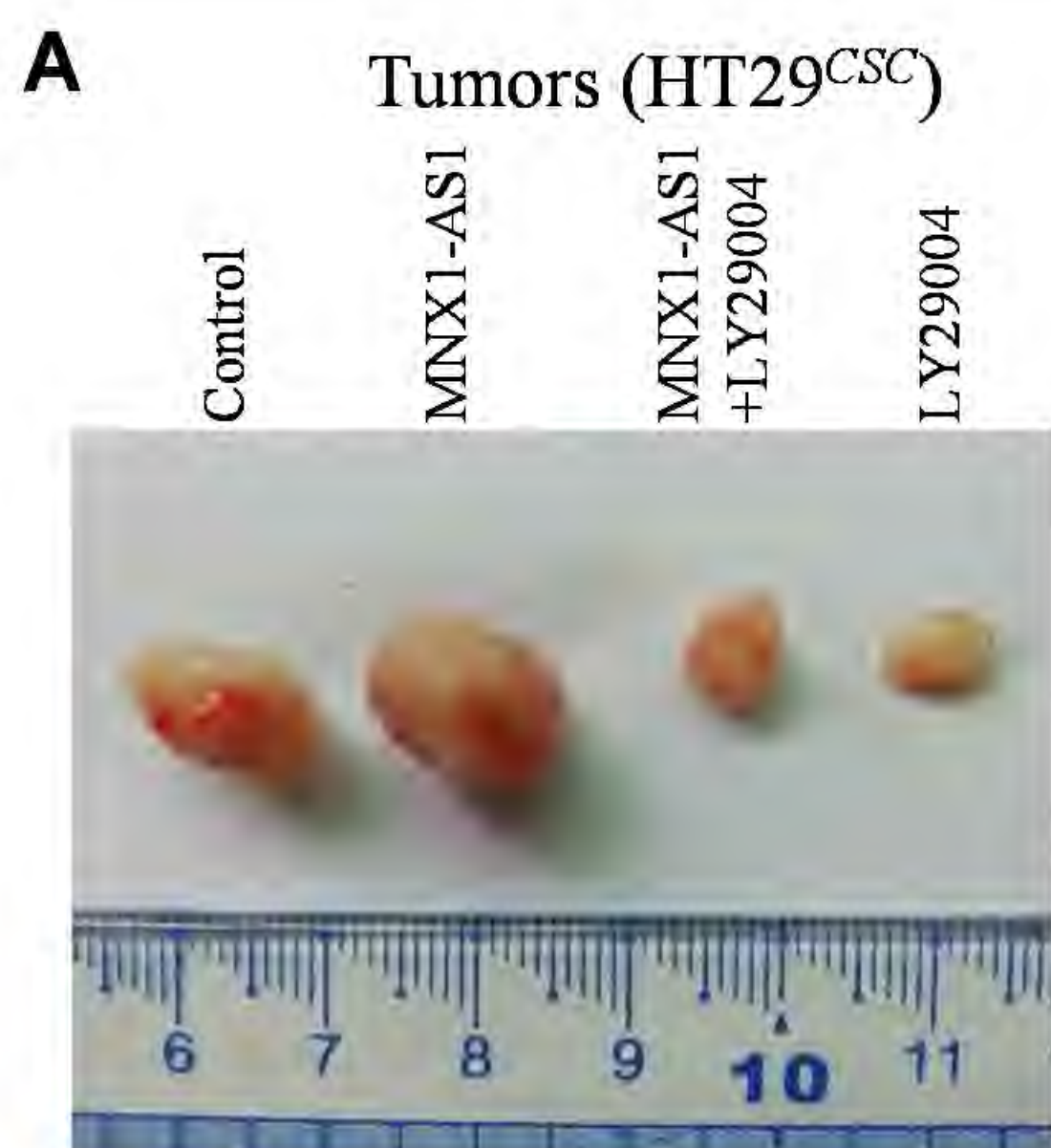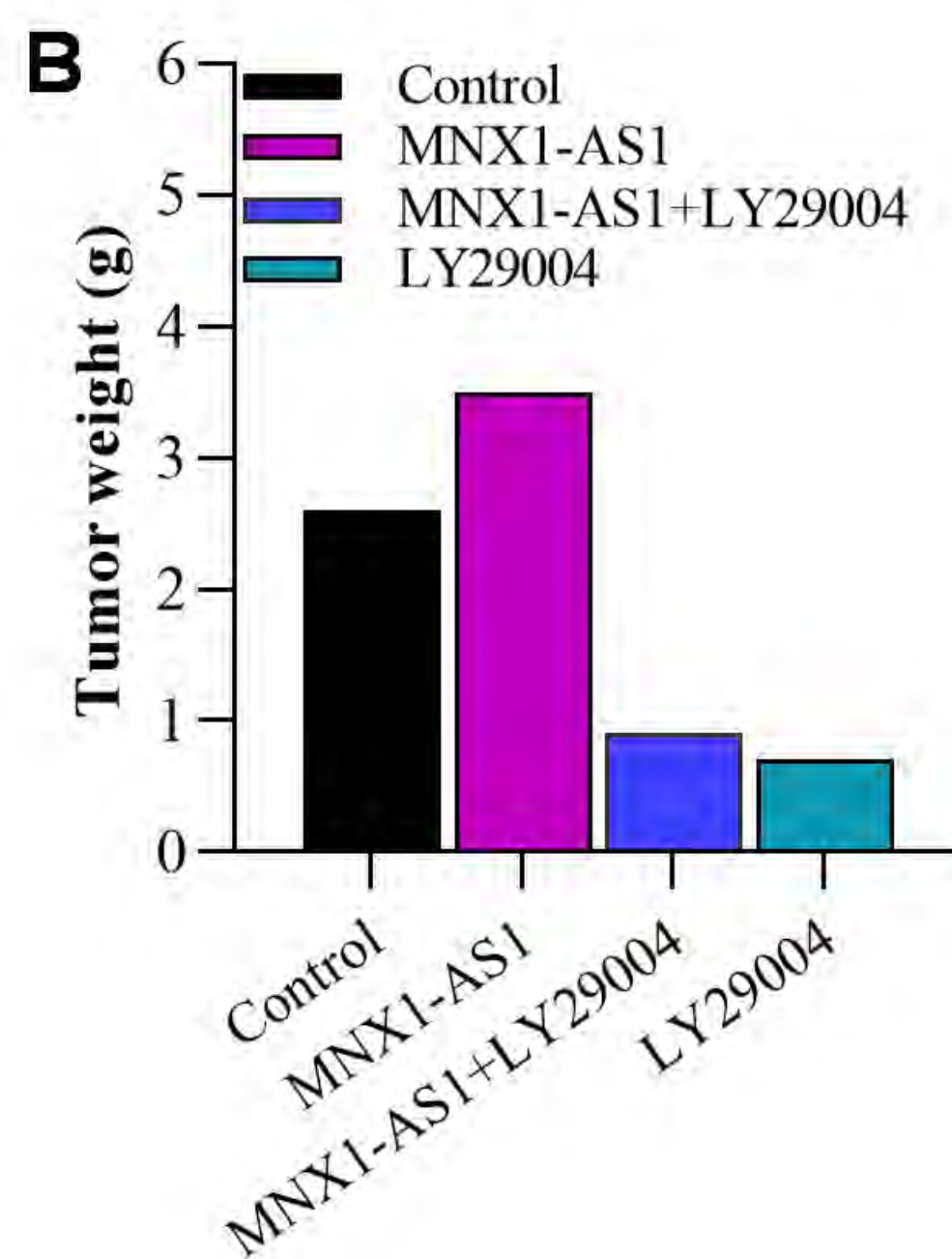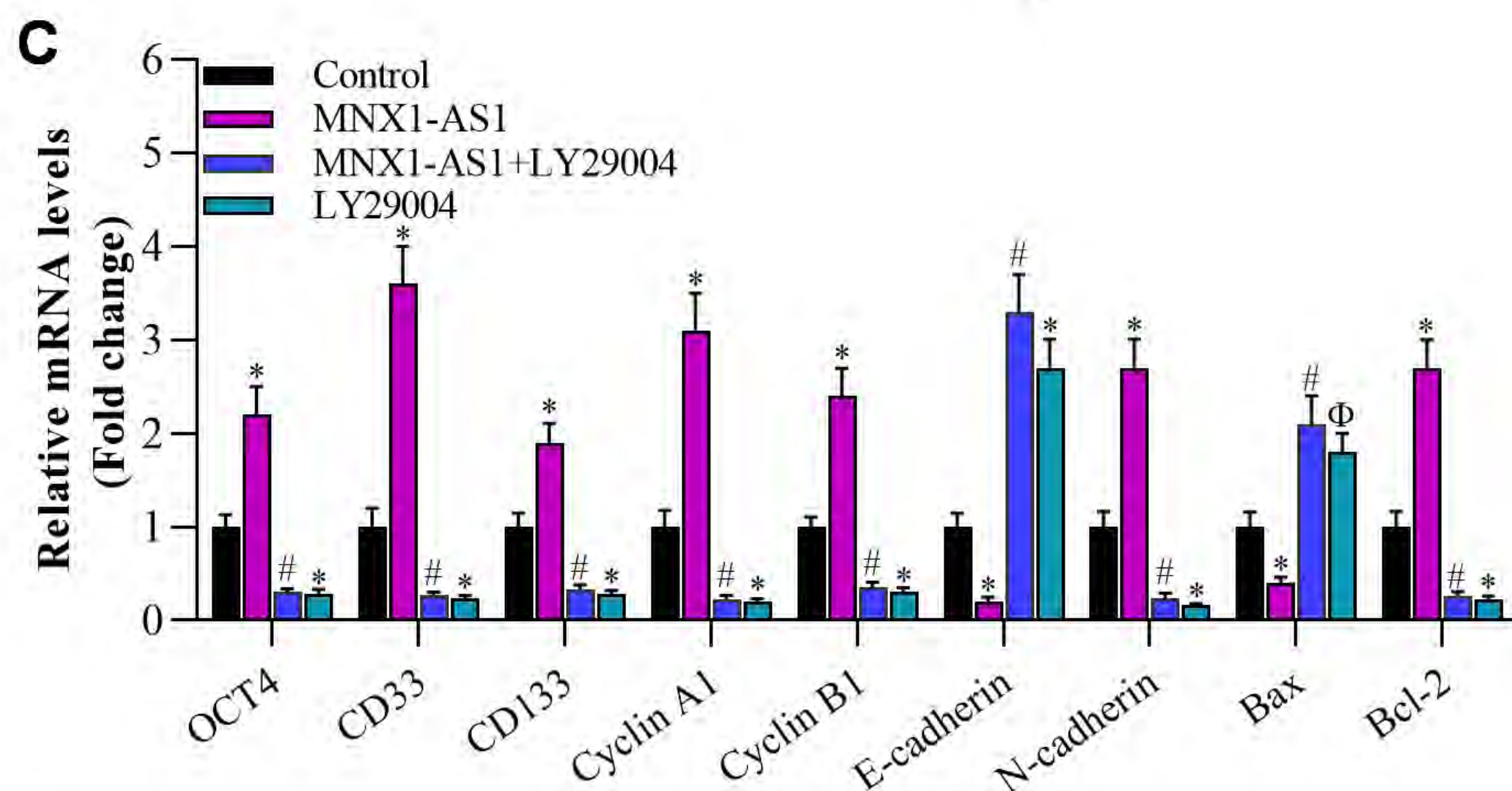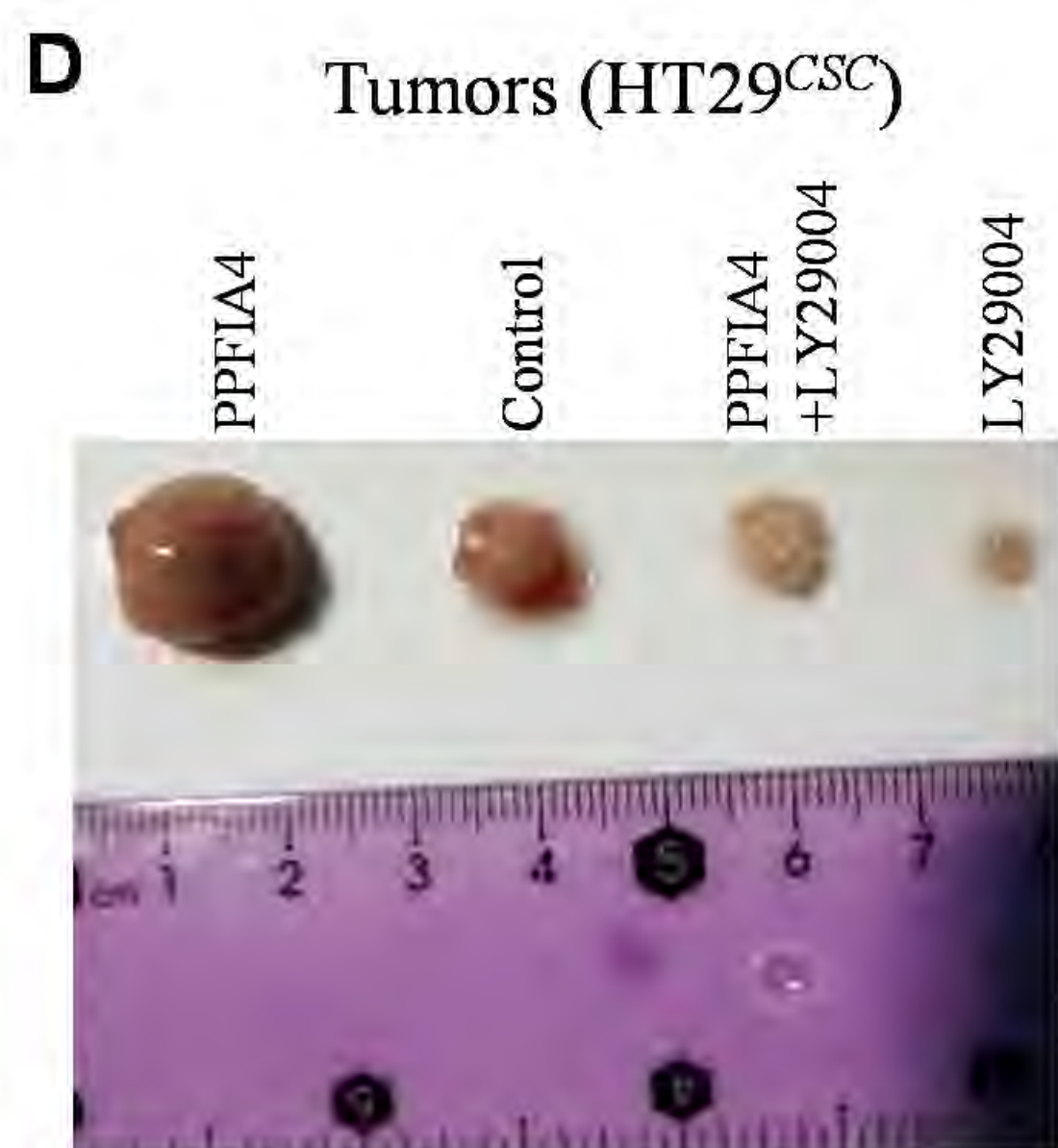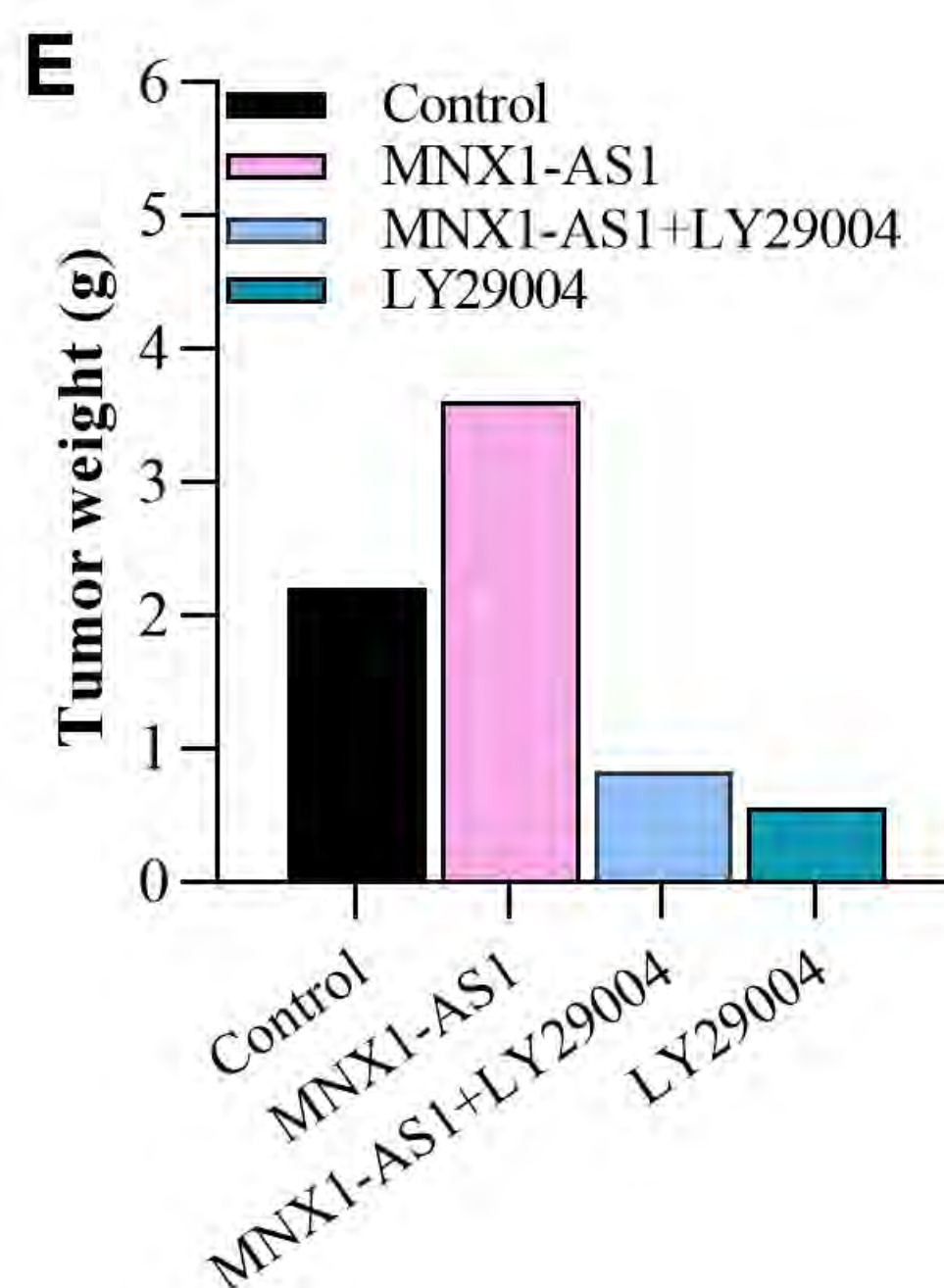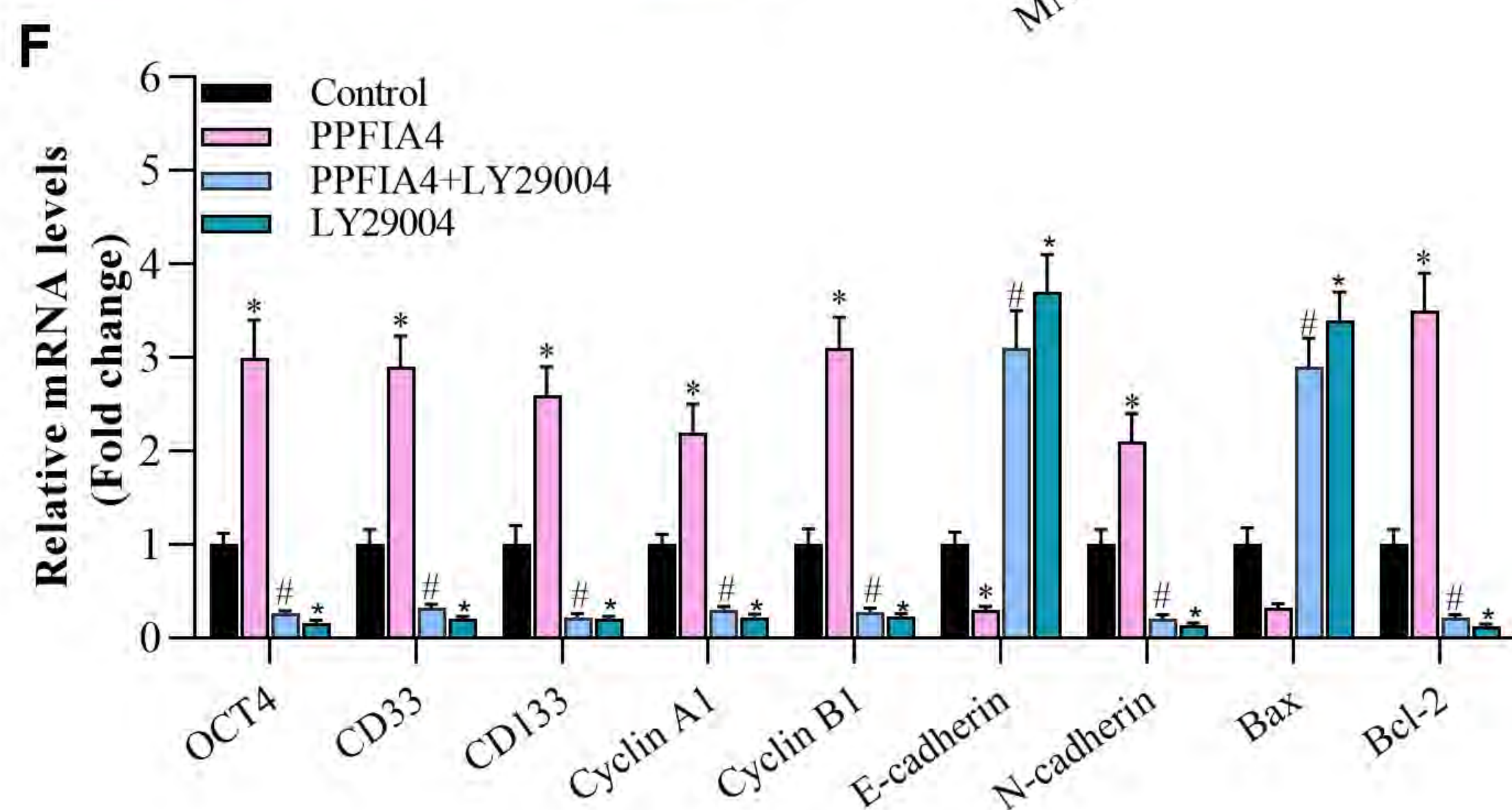

Supplement: Supplementary Materials — Suppl. Figure 1. LY29004 rescues MNX1-AS1 and PPFIA4 in vivo. (a, d) Nude mouse xenograft tumors. (b, e) After 15 days, the tumors were weighed. (c, f) Expression of tumor cell stemness, proliferation, migration, and apoptosis-related genes in tumor tissues of each group, ∗P < 0.05 compared with control group, and #P < 0.05 compared with MNX1-AS1 group or PPFIA4 group. [file 8303409.f1.pdf]
